# The Impact of a Nursing Strike on Glycemic Control in Hospitalized Patients with Diabetes

**DOI:** 10.7759/cureus.16020

**Published:** 2021-06-29

**Authors:** Kelsey H Sheahan, Amanda G Kennedy, Bradley J Tompkins, Allen B Repp, Matthew P Gilbert

**Affiliations:** 1 Endocrinology, Northwestern Medical Center, St. Albans, USA; 2 Larner College of Medicine, University of Vermont, Burlington, USA

**Keywords:** endocrinology and diabetes, inpatient care, medical workforce, nursing staff, diabetes treatment

## Abstract

Introduction

Hyperglycemia and hypoglycemia have been found to increase morbidity and mortality among hospitalized patients with diabetes. In July of 2018, our academic medical center experienced a 48-hour nursing strike, during which time 600 replacement nurses were employed. This cohort study evaluated the impact of the nursing strike on glycemic control among hospitalized patients with diabetes.

Methods

Point-of-care fingerstick blood glucose (POC BG) values among hospitalized patients with diabetes were compared between the 48-hour nursing strike period and two 48-hour periods when the nursing strike did not occur. We evaluated the percentage of POC BG values that were hyperglycemic (POC BG 181-250 mg/dL), severely hyperglycemic (POC BG >250 mg/dL), and hypoglycemic (POC BG <70 mg/dL). Additionally, we assessed the proportion of patients who experienced one or more days of hypoglycemia, hyperglycemia, or severe hyperglycemia.

Results

We found a significant association between the distributions of POC BG test results during the nursing strike; test results more frequently showed hyperglycemia, severe hyperglycemia, or hypoglycemia during the nursing strike than during the control period (p=0.006). There was a significant difference in the days of hypoglycemia, with 7.7% of patients experiencing one or more days of hypoglycemia during the strike period compared with 1.4% of patients during the control period (p=0.03).

Conclusion

Nursing strikes have been employed as a last resort in contract negotiations with hospitals, but they have the potential to significantly affect patient care and safety. Further studies are needed to evaluate these impacts to prepare for future workforce disruptions.

## Introduction

In 2016, 7.8 million patients with diabetes were hospitalized [1]. Hyperglycemia and hypoglycemia are both associated with increased morbidity and mortality among hospitalized patients with diabetes [2], with 32% of these patients experiencing hyperglycemia and 6% having at least one episode of hypoglycemia [3]. Maintaining glycemic control in hospitalized patients with diabetes is reliant not only on adequate medications, but also on services provided by nurses, including nursing care, the correct administration of medications, patient education, and diet.

An increasing proportion of nurses worldwide are joining unions, and nursing unions have occasionally employed strikes during contract negotiations [4,5]. There is scant literature on the impact of these strikes on patients’ glycemic control; however, nurse staffing levels are associated with the timing of insulin administration [6].

In July 2018, the Vermont Federation of Nurses and Health Professionals voted to authorize a 48-hour strike after unsuccessful negotiations with the University of Vermont Medical Center (UVMMC). After receiving advance notice of this strike, UVMMC hired 600 replacement nursing staff who were employed during the 48 hours of the strike [7]. The purpose of this study was to evaluate the impact of the strike on glycemic control among hospitalized patients with diabetes. To our knowledge, this is the first study to evaluate glycemic control during a nursing strike.

This article was previously presented as a meeting abstract at the American Diabetes Association 80th Scientific Session on June 13, 2020.

## Materials and methods

This was a retrospective study of glycemic control before, during, and after a nursing strike at UVMMC. The strike occurred for 48 hours from Thursday, July 12, 2018, to Saturday, July 14, 2018. The control group was a combined group of two 48-hour periods as follows: the first group was 1 month prior to the strike and the second was 1 month following the strike. The timing of each of the two data collection periods was matched to be the second Thursday to Saturday of the month to account for potential confounding variables. This study was approved as an exempt research by the University of Vermont Committee on Human Research (STUDY00000210).

The study analyzed the records of all hospitalized patients with diabetes (type 1 or type 2) on their problem list and hospitalized patients without diabetes (type 1 or type 2) on their problem list who received subcutaneous insulin injections during their hospital stay. Patients were excluded if they were in the intensive care unit, received intravenous insulin, had more than one visit during the strike or non-strike period, or whose visit extended across the strike and non-strike period.

Point-of-care fingerstick blood glucose (POC BG) values were collected in addition to demographic variables. At our institution, the frequency of POC BG for non-critically ill patients is determined by the patient’s oral intake per standardized protocols. Patients on a diet receive POC BG before meals and at bedtime. Patients who are not on a diet receive POC BG every 6 hours.

The primary endpoint was the percentage of hyperglycemic POC BG readings during the 48 hours, defined as the number of POC BG values between 181 and 250 mg/dL divided by the total number of POC BG values. The secondary endpoints included the percentage of severely hyperglycemic POC BG readings (defined as the number of POC BG values >250 mg/dL divided by the total number of POC BG values), percentage of hypoglycemic POC BG readings (defined as the number of POC BG values <70 mg/dL divided by the total number of POC BG values), number of POC BG tests performed per 24 hours, and the median POC BG level. As the hyperglycemia and hypoglycemia percentages could be affected by differences in the frequency of repeated measurements of abnormal values, we also calculated differences in the proportion of patients experiencing at least one event of hyperglycemia, severe hyperglycemia, or hypoglycemia per day (days of hyperglycemia, severe hyperglycemia, and hypoglycemia, respectively) during the strike period and control periods.

Statistical analyses were conducted using Wilcoxon rank-sum tests for continuous variables and the chi-square or Fisher’s exact tests for categorical variables. Differences were considered statistically significant at a p-value of < 0.05. Analyses were conducted using STATA 16.1 (Stata Corporation, College Station, TX, USA).

## Results

A total of 211 patients with diabetes were included in the analysis: 65 (30.8%) and 146 (69.2%) patients were hospitalized during the strike and control periods, respectively. There were no significant differences between the baseline characteristics of the strike and control groups with regard to sex, race, age, or body mass index (Table 1).

**Table 1:** Characteristics of hospitalized patients with diabetes or requiring insulin Values are presented as n (%) or median (interquartile range)

Category	Strike period (n=65)	Control period (n=146)	p
Sex (female)	31 (47.7)	60 (41.1)	0.372
Race (Caucasian)	62 (96.9)	137 (95.8)	0.712
Age	68.0 (59–74)	67.5 (58–78)	0.793
Body mass index	30.4 (26.3–34.6)	30.7 (26.6–37.6)	0.445

Among the 211 patients, a total of 1,225 POC BG tests were evaluated: 413 (33.7%) from the strike period and 812 (66.3%) from the control period. A significantly higher number of POC BG tests were performed daily during the strike period (4.5 per patient per day) than during the control period (4.1 per patient per day, p=0.038).

The proportion of test results that were within the normal range, hyperglycemic, severely hyperglycemic, or hypoglycemic differed significantly between the strike and control periods (Table 2, p=0.006). Within the strike period, test results more often showed hyperglycemia (27.9% vs. 22.7% in the control period), severe hyperglycemia (12.1% vs. 9.1%), and hypoglycemia (1.5% vs. 0.5%).

**Table 2:** Glucose test results during the strike and control periods The distribution of glucose test results across the four parameters differed significantly between the strike and control periods (Fisher’s exact test, p =0.006).

	Strike period (n =413)		Control period (n=812)	
	n	(%)	n	(%)
Normal range (70–80 mg/dL)	242	(58.6)	550	(67.7)
Hyperglycemic (181–250 mg/dL)	115	(27.9)	184	(22.7)
Severely hyperglycemic (>250 mg/dL)	50	(12.1)	74	(9.1)
Hypoglycemic (<70 mg/dL)	6	(1.5)	4	(0.5)

There were no significant differences in the proportion of patients who experienced one or more days of hyperglycemia (56.9% vs. 52.7%, p=0.57) or severe hyperglycemia (27.7% vs. 23.3%, p=0.49) between the strike and control periods. However, the proportion of patients who experienced one or more days of hypoglycemia was significantly greater during the strike period than during the control period (7.7% vs. 1.4%, p=0.03) (Table 3). The median POC BG value was significantly higher during the strike period (164 mg/dL) than during the control period (150 mg/dL, p≤0.01) (Table 4).

**Table 3:** Proportion of patients who experienced one or more days of hyperglycemia (>180 mg/dL), severe hyperglycemia (>250 mg/dL), or hypoglycemia (<70 mg/dL) during the strike and control periods

	Strike period (n =413)		Control period (n=812)		p
	n	(%)	n	(%)	
Hyperglycemia ≥ 1 day	37	(56.9)	77	(52.7)	0.573
Severe hyperglycemia ≥ 1 day	18	(27.7)	34	(23.3)	0.493
Hypoglycemia ≥ 1 day	5	(7.7)	2	(1.4)	0.030

**Table 4:** Secondary analyses including the frequency of glucose tests and median glucose level Values are presented as median (interquartile range)

Patient-level measures	Strike Period		Control Period		p
Glucose tests/24 hours	4.5	(3.9–5.5)	4.1	(3.3–4.9)	0.038
Glucose level (mg/dL)	164	(127–207)	150	(123–193)	<0.007

## Discussion

Our study found a significant association between the distribution of POC BG test results and the nursing strike; test results more frequently showed hyperglycemia, severe hyperglycemia, and hypoglycemia during the strike period. However, when considering the proportion of patients with at least one day of hyperglycemia, severe hyperglycemia, or hypoglycemia, there was a significant difference only regarding the days of hypoglycemia, with 7.7% of patients in the strike group experiencing at least one day of hypoglycemia versus 1.4% of patients in the control group. Although the median POC BG value was significantly higher during the strike period than during the control period (164 mg/dL vs. 150 mg/dL), both of these values fall within the goal POC BG range for hospitalized patients with diabetes according to the guidelines of the American Diabetes Association [8].

Maintaining good glycemic control in hospitalized patients with diabetes is important [2]. Glycemic control depends not only on correct insulin dosing, but also on the coordination of insulin delivery and food intake, correct timing of insulin administration, correct injection technique for insulin, and other factors [9]. Because many of the important aspects of glycemic control are performed by the inpatient nursing staff, nursing strikes have the potential to adversely affect glycemic control.

The percentage of hypoglycemic results was significantly higher during the strike period (1.4% vs. 0.5% during the control period), and significantly more patients experienced one or more days of hypoglycemia during the strike. Hypoglycemia has many potential causes, making it difficult to elucidate the exact mechanism of these findings. Potential contributors include inadequate coordination of insulin delivery and meal delivery, a common risk factor for hypoglycemia [10], as well as unfamiliarity with meal delivery protocols and hospital-specific protocols for administering insulin. However, we were unable to ascertain whether the scheduled pre-meal insulin was administered within the appropriate window after meal delivery because our institution allows meals to be delivered at the request of the patient, and delivery times are not recorded in the electronic medical record. We believe that this is an important area for future research. Additionally, hospitals typically employ one delivery device for insulin. While most hospitals use insulin pens, some institutions may use multi-dose insulin vials or a combination of both modalities. There are reports in the literature on medication errors associated with transitioning from one delivery system to another [11], and it is possible that the replacement nurses could have been unfamiliar with the preferred insulin delivery method at our institution. Instructions regarding the use of specific insulin pens were not included in the initial onboarding of the replacement nurses.

This study also found that POC BG values were checked more frequently during the strike period, which could have amplified the percentages of hyperglycemia, severe hyperglycemia, and hypoglycemia readings, particularly given that our institution’s hypoglycemia protocol requires repeated blood glucose monitoring following an episode of hypoglycemia to ensure the patient’s blood sugar has normalized. A lack of familiarity with the institution’s several policies regarding the care of patients with diabetes and/or unique aspects of the delivery of nutritional services at our institution could also have accounted for the increased amount of blood glucose testing during the strike period. However, we also examined the proportion of patients experiencing one or more days of hyperglycemia, severe hyperglycemia, and hypoglycemia during the strike compared with the control period to mitigate the effects of testing frequency, which should have theoretically eliminated this confounder.

Despite recommendations for optimizing the care of hospitalized patients with diabetes, significant variability remains among institutions regarding the availability of resources such as inpatient endocrinology consultation, inpatient certified diabetes educators, nurse clinicians, and clinical protocols to effectively care for these patients. The introduction of staff shortages, or in the case of our institution, a nursing strike, could greatly impact the quality of care for patients with diabetes.

One of the limitations of our study was its retrospective design. Second, the study was conducted in one academic institution with specific insulin and glycemic protocols. Third, there was a potential for unmeasured differences between the patients hospitalized during the strike and control periods. We did not adjust for potential confounding of other important variables, such as concomitant medications or potential variability in staffing numbers. Despite these limitations, our findings support the importance of well-trained nursing staff in the care of hospitalized patients with diabetes. We recommend that onboarding procedures for replacement nurses or traveling nurses be standardized to include a complete and detailed review of all institutional policies regarding the care of patients with diabetes, including, but not limited to, the use of the insulin pen delivery device, treatment of hypoglycemia and hyperglycemia, nutritional policies regarding meal delivery, and educational resources available to bedside nurses.

## Conclusions

Nursing strikes are a known tool in negotiations between hospitals and nurses. Our study found a significant association between the strike period and an increased percentage of hypoglycemic blood glucose readings and an increased proportion of patients experiencing at least one or more days of hypoglycemia during the strike period. This study aims to stimulate conversations among institutions on how to care effectively for patients with diabetes during workforce disruptions. Additional studies are needed to examine patient care and safety during nursing strikes in order to identify specific areas of clinical care that should be emphasized in the training and onboarding of traveling nurses or replacement nurses during staffing shortages or nursing strikes.

## References

[1] (2021). Centers for disease control and prevention. National diabetes statistics report, 2020. Estimates of diabetes and its burden in the United States. https://www.cdc.gov/diabetes/pdfs/data/statistics/national-diabetes-statistics-report.pdf.

[2] Clement S, Braithwaite SS, Magee MF (2004). Management of diabetes and hyperglycemia in hospitals. Diabetes Care.

[3] Swanson CM, Potter DJ, Kongable GL, Cook CB (2011). Update on inpatient glycemic control in hospitals in the United States. Endocr Pract.

[4] Youssef NA, Mostofi MB, Barnewolt BA, Youssef R, Weiner SG (2021). The effect of a nursing strike on emergency department operational metrics. Am J Emerg Med.

[5] Leskovan JJ, Pahl J, Stringfellow K (2021). The impact of clinical crisis management on trauma team outcomes following a prolonged strike by nursing, technical, and support staff. J Healthc Risk Manag.

[6] Ng JM, Narayanan D, Mellor DD (2010). Ward staffing levels significantly affect timing of insulin administration in hospital. Practical Diabetes.

[7] (2021). Nurses picket as strike starts at UVM medical center. seven days. https://www.sevendaysvt.com/OffMessage/archives/2018/07/12/slideshow-nurses-picket-as-strike-starts-at-uvm-medical-center.

[8] American Diabetes Association (2021). 15. Diabetes care in the hospital: standards of medical care in diabetes-2021. Diabetes Care.

[9] (2021). Institute for safe medication practices. ISMP guidelines for optimizing safe subcutaneous insulin use in adults. https://www.ismp.org/sites/default/files/attachments/2017-11/ISMP138-Insulin%20Guideline-051517-2-WEB.pdf.

[10] Hulkower RD, Pollack RM, Zonszein J (2014). Understanding hypoglycemia in hospitalized patients. Diabetes Manag.

[11] Trimble AN, Bishop B, Rampe N (2017). Medication errors associated with transition from insulin pens to insulin vials. Am J Health Syst Pharm.

